# Poly(ε-caprolactone)-poly(ethylene glycol) Tri-Block Copolymer as Quercetin Delivery System for Human Colorectal Carcinoma Cells: Synthesis, Characterization and In Vitro Study

**DOI:** 10.3390/polym15051179

**Published:** 2023-02-26

**Authors:** Nancy Ferrentino, Maria Preziosa Romano, Silvia Zappavigna, Marianna Abate, Vitale Del Vecchio, Dario Romano, Chiara Germinario, Celestino Grifa, Rosanna Filosa, Daniela Pappalardo

**Affiliations:** 1 Dipartimento di Scienze e Tecnologie, Università del Sannio, Via de Sanctis snc, 82100 Benevento, Italy; 2 Advanced Medical Pharma, (AMP-BIOTEC) Healthcare Research and Innovation Center, 82030 San Salvatore Telesino, Italy; 3 Department of Precision Medicine, University of Campania “Luigi Vanvitelli”, Via Santa Maria di Costantinopoli, 16, 80138 Naples, Italy; 4 Department of Experimental Medicine, University of Campania “Luigi Vanvitelli”, Via Santa Maria di Costantinopoli, 16, 80138 Naples, Italy; 5 Physical Science and Engineering Division, King Abdullah University of Science and Technology (KAUST), Thuwal 23955-6900, Saudi Arabia; 6 Istituti Clinici Scientifici Maugeri IRCCS, Cardiac Rehabilitation Unit of Telese Terme Institute, 82037 Telese Terme, Italy

**Keywords:** polycaprolactone-*block*-polyethylenglycol, copolymers, quercetin, drug delivery system, colorectal carcinoma cells

## Abstract

Quercetin is a hydrophobic molecule with short blood circulation times and instability. The development of a nano-delivery system formulation of quercetin may increase its bioavailability, resulting in greater tumor suppressing effects. Triblock ABA type polycaprolactone-polyethylenglycol- polycaprolactone (PCL-PEG-PCL) copolymers have been synthetized using ring-opening polymerization of caprolactone from PEG diol. The copolymers were characterized by nuclear magnetic resonance (NMR), diffusion-ordered NMR spectroscopy (DOSY), and gel permeation chromatography (GPC). The triblock copolymers self-assembled in water forming micelles consisting of a core of biodegradable polycaprolactone (PCL) and a corona of polyethylenglycol (PEG). The core-shell PCL-PEG-PCL nanoparticles were able to incorporate quercetin into the core. They were characterized by dynamic light scattering (DLS) and NMR. The cellular uptake efficiency of human colorectal carcinoma cells was quantitatively determined by flow cytometry using nanoparticles loaded with Nile Red as hydrophobic model drug. The cytotoxic effect of quercetin-loaded nanoparticles was evaluated on HCT 116 cells, showing promising results.

## 1. Introduction

The development of delivery systems for anticancer drugs into tumor tissues to avoid systemic toxicity is a crucial challenge in cancer therapy. Drug delivery systems (DDSs) could increase the therapeutic effect, the bioavailability, circulation time, and stability of drugs [1,2,3,4,5,6]. Several DDSs have been investigated including polymers, liposomes, and block copolymer micelles [7]. Compared to liposomes, polymeric nanoparticles, have a greater stability in vivo and they are more versatile, since polymer structure, molecular weights, and composition can be changed to meet the requirements of a particular application [8]. Polymeric nanoparticles based on amphiphilic block copolymers have aroused significant interest because of their ability to self-assemble into a micellar form in water phase, with a hydrophilic outer shell or corona and a hydrophobic core. The hydrophilic shell of the micelle prevents steric recognition by the immune system and its removal from the bloodstream by the macrophage system. At the same time, the hydrophobic core can encapsulate fragile hydrophobic anticancer therapeutics [9,10]. The encapsulation of anticancer drugs in nanocarriers enhances drug accumulation in the tumor tissues through the enhanced permeability and retention (EPR) effect [1].

Polymeric nanoparticles derived from biodegradable and biocompatible polyesters have been largely used as a DDS. Many studies have been conducted on micelles consisting of a core of biodegradable polycaprolactone (PCL) and a corona of polyethylenglycol (PEG). PEG is hydrophilic and it exhibits excellent biocompatibility and lack of toxicity. The presence of PEG as an outer shell in DDSs reduces opsonization and the aggregation of DDSs in vivo [11]. PCL is non-toxic and biodegradable; these characteristics are important for drug delivery as they help maintain clearance of the nanocarrier after injection. PCL-based systems can be completely degraded within the body due to the presence of ester bonds that can easily be subjected to hydrolysis by esterases [12]. The main degradation product of PCL is 6-hydroxycaproic acid, that can be eliminated by urinary excretion or pulmonary elimination as carbon dioxide [13]. Due to the high degree of crystallinity and hydrophobicity, PCL is less biocompatible with soft tissues limiting its clinical application. Therefore, the addition of PEG to the PCL chain allows improvements to the hydrophilicity, biodegradability, and performance in the cell culture studies when compared with the PCL homopolymer, thus allowing the use of such systems in different biomedical applications [14]. 

The synthesis of PCL–PEG block copolymers can be achieved by the ring-opening polymerization of ε-caprolactone (ε-CL) using PEG as initiator [10,15]. The molecular weight of the PCL block is primarily controlled by the molar ratio of ε-CL to the initiator PEG. Copolymers of various block lengths of PEG and PCL have been described in the literature. Moreover, diblock (AB) or triblock (ABA) copolymers have been prepared using a mono-hydroxy or α, ω-dihydroxy PEG as initiator for the polymerization of lactone monomers [14,16,17,18,19].

PEG-PCL-based DDSs have been used as carriers of diverse hydrophobic drugs and they have been shown to induce therapeutic effects both in vitro and in vivo. Many in vitro toxicity studies have been performed on PEG-PCL nanoparticle systems. These systems proved to be stable and non-toxic, allowing them to protect the drug and to administer it in a controlled way. To mention some of them, nanoparticles constituted by diblock PEG-PCL were tested in vitro and in vivo for the delivery of paclitaxel with high encapsulation efficiency for glioblastoma multiform treatment [20]. Doxorubicin-loaded nanoparticles made of triblock PCL-PEG-PCL were proposed as promising DDSs for breast cancer therapy [21]. Moreover, nanoparticles based on the same PCL-PEG-PCL triblock copolymer were able to simultaneously encapsulate two drugs, doxorubicin and quercetin [22]. High drug-loading efficiency and efficient cellular uptake were also recognized for the PTX-loaded PCL-PEG-PCL nanoparticles in EMT-6 breast cancer cells [23].

PEG-PCL drug delivery systems have also been tested on colon cancer cells. Nanoparticles based on the PEG-PCL-PEG triblock copolymer conjugated with folic acid and loaded with 5-fluorouracil and magnetite increased the inhibitory effect against several colon cancer cells when compared with free drug applications [24]. Moreover, PCL-PEG diblock copolymers have been coupled to tyrosine and angiopep-2 to obtain docetaxel-loaded PEG-PCL-dual-modified-nanoparticles for the treatment of colorectal cancer [25]. Di-block PEG-PCL copolymers were an important DDS for tetradrine delivery [26], while triblock PCL-PEG-PCL copolymers, loaded with auraptene, increased drug biodistribution and bioavailability into colon cancer cells [27].

All these results indicated that PEG-PCL nanoparticles had great potential as optimal delivery systems for hydrophobic molecules and to increase the pharmacokinetic profile of drug but also to decrease the metabolism of active compound. Taking this paradigm into account, the combination of natural products with anticancer properties and chemotherapy can be a novel strategy for both the possible improvement of therapeutic efficacy of drugs and in achieving better pharmacokinetic profiles and avoiding the side effects of drug toxicity. For several decades, flavonoids played an essential role in the human diet as antioxidants, antinflammatories, and cancer/chemo preventive agents [28]. Among those known for chemo-preventive efficacy, quercetin, as a member of the flavonols subgroup, is one of the main compounds that has been extensively described in several in vitro and in vivo studies [29]. In this regard, its beneficial anti-mutagenic and anti-proliferative effects, its antioxidant properties, and its role in the regulation of cell signalling, cell cycle, and apoptosis, assert its significant pharmacological potential against cancer. Unfortunately, the bioavailability of quercetin is well known to be poor in limited low plasma concentrations. It is reported that the application of a nanosystem formulation of quercetin results in an increased water solubility and thus enhancement of its bioavailability, resulting in greater tumor suppressing effects [30,31].

In the framework of our interest in block copolymers based on PEG and resorbable aliphatic polyesters [32,33,34,35], we have synthetized and characterized amphiphilic PCL-PEG-PCL triblock copolymers and obtained micelle nanoparticles. The micelles were loaded with the hydrophobic molecule Nile Red (NR) to characterize and evaluate the loading capacity of the nanoparticles and monitoring the entry and loading of the fluorescent substance. Subsequently, the micelles were loaded with quercetin to confirm their capability to maintain the anticancer property of the natural compound, increasing its water solubility.

## 2. Materials and Methods

### 2.1. Instruments and Measurements

#### 2.1.1. Nuclear Magnetic Resonance (NMR)

NMR spectra were recorded on a Bruker AM300 (^1^H, 300 MHz; ^13^C, 75 MHz) and a Bruker Advance 400 (^1^H, 400 MHz; ^13^C, 100 MHz). Chemical shifts (^1^H and ^13^C NMR) were assigned using tetramethylsilane (TMS) as an internal reference and were expressed as parts per million. Coupling constants (J) were expressed in Hertz. NMR spectra were referenced using the residual solvent peak at δ = 7.27 (^1^H) for CDCl_3_ and at δ = 2.50 (^1^H) for DMSO. NMR signals were reported as follows: chemical shift (δ ppm), relative integral, and multiplicity (s = singlet, d = doublet, t = triplet, q = quartet, m = multiplet, dd = doublet of doublet, br = broad). The NMR samples were prepared by dissolving about 10 mg of the compound in 0.5 mL of the deuterated solvent. Spectra were recorded using Bruker TopSpin v3.2 software. Data processing was performed using TopSpin v3.2 or MestReNova v12.0.2 software.

2D Diffusion-Ordered (DOSY) PGSE NMR spectra of the block copolymers were recorded on a Bruker Avance 400 spectrometer 10 mg of the copolymer were dissolved in 0.5 mL of CDCl_3_ and the spectra were recorded at room temperature without spinning; the parameters δ and Δ were kept constant during the experiments, whereas G was varied from 2 to 95 % in 25 steps, 64 scans per step.

#### 2.1.2. Gel Permeation Chromatography (GPC)

Gel permeation chromatography was performed using a GPC Agilent with a refractive detector and a PLgel 5 µm Mixed-C column. The samples were dissolved and eluted in tetrahydrofuran. After complete dissolution, samples were filtered through PTFE membranes (0.22 µm). The injection volume was 20.00 µL and the flow rate was 1.00 mL/min. The chosen method of analysis was universal calibration based on polystyrene standards with a narrow molecular weight distribution. The measurements were performed at 35 °C according to the temperatures of the columns and detectors.

#### 2.1.3. Fourier Transform Infrared Spectroscopy (FTIR)

FTIR analyses were carried out with the diamond crystal Attenuated Total Reflectance module (ATR) of a Bruker ALPHA spectrometer, equipped with ROCKSOLID™ interferometer and a ZnSe/KBr beam splitter with a DTGS detector. The spectra, processed using Bruker OPUS 7.2 software, were acquired in the mid-infrared spectral range (4000–400 cm^−1^) with a resolution 4 cm^−1^ and 64 scans per minute.

#### 2.1.4. Dinamic Light Scattering (DLS)

DLS measurements were performed using a Malvern Zetasizer Nano Z. Measurements were performed at 25 °C and at 37 °C after an equilibration time of 60 s. The angle of detection was 173° using the non-invasive back scatter system. The measurements were performed in deionized water; the viscosity (η) and the refractive index (RI) of the dispersant at 25 °C (η_25_ = 0.8960 mPa s; RI_25_ = 1.393) and 37 °C (η_37_ = 0.7424 mPa s; RI_37_ = 1.391) were set accordingly. 

The intensity-weighted mean values of the diameter and polydispersity index (PDI) were reported as the average of three measurements. For each measurement, 10 scans were recorded and the reported error represents the average error incurred during the three measurements.

The quercetin-loaded nanoparticles (0.78 mg) were suspended in deionized water (2 mL), sonicated for 30 min, and then placed in a glass cuvette and analyzed.

### 2.2. Chemicals

Moisture and air-sensitive materials were manipulated under nitrogen using Schlenk techniques. The chemicals anhydrous toluene (99.8%), hexane (≥95%), stannous octoate (92.5–100.0%) (SnOct_2_), methanol (99.8%), dimethylformamide (≥99.8%) (DMF), chloroform (≥99.5%), tetrahydrofuran (≥99.9%) (THF), Nile Red (≥97.0%) (NR), quercetin (≥95%) (Q), and 3-(4,5-dimethylthiazol-2-yl)-2,5-diphenyltetrazolium bromide (≥97.5%) (MTT) were acquired from Merck and used as received. The ε-caprolactone (97%) (ε-CL), acquired from Merck, was distilled in vacuo from CaH_2_ prior to use. Polyethylene glycol (PEG-2000 MW), acquired from Merck, was dried in vacuo over phosphorus pentoxide (P_2_O_5_) for 72 h.

### 2.3. Cell Cultures

HCT-116 and LoVo cells, both human colon carcinoma cells (American Type Tissue culture Collection, Rockville, MD, USA), were cultured in Dulbecco’s Modified Eagle Medium (DMEM) and F-12K, respectively, and both supplemented with 10% heat inactivated Fetal Bovine Serum (FBS), 100 μg/mL penicillin, 100 μg/mL streptomycin, 1% L-glutamine, and 1% sodium pyruvate. Cells were grown at 37 °C in a humidified atmosphere of 95% air/5% CO_2_.

### 2.4. Synthetic Procedures

#### 2.4.1. Synthesis of Triblock PCL-PEG-PCL Copolymer

The PCL-PEG-PCL triblock copolymer was synthesized by ring-opening polymerization (ROP) of ε-CL on PEG and in the presence of stannous octanoate as catalyst under nitrogen atmosphere. In detail, 0.5 g of telechelic PEG-2000 (0.25 mmol) and ε-CL (2.2 mL, 19.8 mmol) were introduced in a three neck flask and then 6.1 mL of a 9 mM solution of Sn(Oct)_2_ in toluene was added. The resulting mixture was placed in an oil bath at 130 °C and stirred for 44 h. After that time, the mixture was cooled to room temperature, dissolved in chloroform, and the resulting mixture was poured in hexane. The precipitated triblock copolymer was recovered by filtration and dried in vacuo. Yield: 96%.

^1^H NMR (400 MHz, CDCl_3_): δ = 4.04 (t, 2H, -(CH_2_)_4_CH_2_O-), 3.65 (s, 4H,-OCH_2_CH_2_O-), 2.29 (t, 2H, -CH_2_C(O)O-), 1.64–1.60 (m, 4H, -CH_2_-), 1.37 (m, 2H, -CH_2_-).

*M_n_*_NMR_ = 11,020 g/mol; GPC analysis (THF): *M_n_* = 19,420 g/mol; *Đ* = 1.9

#### 2.4.2. Preparation of Triblock PCL-PEG-PCL Copolymer Nanoparticles (NP)

The PCL-PEG-PCL copolymer (10 mg) was dissolved in 1.0 mL of DMF then transferred into 10 mL of stirred deionized water at room temperature. After 2 h, the solution was added to a dialysis membrane with a molecular weight cutoff between 6000 and 8000 g/mol and a volume per length ratio of 1.7 mL cm^−1^ then dialyzed against water for 72 h. The dialysis solution was replaced with fresh deionized water every 3 h; then, dialysis was allowed to occur overnight. The solution of the purified nanoparticles was then lyophilized. 

#### 2.4.3. Preparation of Triblock PCL-PEG-PCL Copolymer Nanoparticles Loaded with Nile Red (NP+NR)

The PCL-PEG-PCL copolymer (50 mg) was dissolved in 5 mL of DMF and a solution of Nile Red (1.5 mg, 4.7 μmol) in 1.5 mL of DMF was slowly added. The obtained solution was transferred into 10 mL of deionized water and stirred for 2 h at room temperature. After this time, the solution was dialyzed against 3 L of deionized water for 72 h with water changes every 3 h. The NR-loaded micelles were dried by using lyophilization.

The loading of NR was evaluated by ^1^H NMR measuring the molar amount of NR in respect to the molar amount of the copolymer using the following formula:

NR content (% *w*/*w*) = MW_NR_ × mol_NR_/(MW_copolymer_ × mol_copolymer_ + MW_NR_ × mol_NR_) × 100 = 1.6%

The loading efficiency was evaluated by ^1^H NMR using the following formula: % loading efficiency = [w_(NR in NP)_]/[w_(initial NR added)_] × 100 = 53%

#### 2.4.4. Preparation of Triblock PCL-PEG-PCL Copolymer Nanoparticles Loaded with Quercetin (NP+Q)

The PCL-PEG-PCL copolymer (10 mg) was dissolved in 1.5 mL of DMF and a solution of quercetin (3 mg, 10 μmol) in 0.5 mL of DMF was slowly added. The obtained solution was transferred into 10 mL of deionized water and stirred for 2 h at room temperature. After this time, the solution was dialyzed against 3 L of deionized water for 72 h. The dialysis solution was replaced with fresh deionized water every 3 h. The quercetin-loaded nanoparticles were dried by using lyophilization.

The loading of quercetin into the nanoparticles was evaluated by ^1^H NMR measuring the molar amount of quercetin respect to the molar amount of the copolymer using the following formula:

Quercetin content (% *w*/*w*) = MW_quercetin_ × mol_quercetin_/(MW_copolymer_ × mol_copolymer_ + MW_quercetin_ × mol_quercetin_) × 100 = 1.6%

The loading efficiency was evaluated by ^1^H NMR by using the following formula: % loading efficiency = [w_(quercetin in NP)_]/[w_(initial quercetin added)_] × 100 = 5.3%

### 2.5. In Vitro Cells Studies

#### 2.5.1. Analysis of Cell Uptake of Nile Red-Loaded Nanoparticles (NP+NR) by Fluorescence Microscopy and Fluorescence-Activated Cell Sorting (FACS)

Nile Red (NR)-loaded nanoparticles PCL-PEG-PCL (NP+NR) were prepared using the dialysis method. HCT 116 e LoVo cells were seeded in 12-well plates at a density of 1 × 10^5^/well and incubated at 37 °C for 24 h. The culture medium containing free NR or NP+NR (10 μM of NR) was added to the cells and incubated for 1 h, 3 h, and 5 h. After incubation, the cells were washed with phosphate-buffered saline (PBS) at pH 7.4 for 1 min and observed under the fluorescence microscope. Red fluorescence was observed by an inverted digital fluorescence microscope (EVOS M5000 Imaging System, Thermo Fisher Invitrogen) using a 20× magnification.

A BD FACSCanto™fluorescent-activated flow cytometer and the BD FACSCanto™ Clinical software (BD Biosciences, San Jose, CA, USA) were used to perform flow cytometry analysis. In detail, the cells were detached with 1X trypsin, centrifuged for 5 min, washed with PBS, centrifuged for 5 min, and fixed with formaldehyde solution (4% *v*/*v*) for 15 min at 4 °C. Then, the cells were washed again with PBS at pH 7.4 and, for each tube, 20,000 cells were measured on a FACSCanto™ flow cytometer (Becton Dickinson, BD Biosciences, San Jose, CA, USA) using FACSCanto™ Clinicalsoftware (Becton Dickinson, BD Biosciences, San Jose, CA, USA). The detection wavelengths were 546 nm and 590 nm for excitation and emission, respectively; the emission spectrum was collected by FLH-2 channel of the FACSCanto™.

#### 2.5.2. Cell Proliferation Assay

Cell proliferation of HCT 116 was evaluated in the presence of quercetin-loaded nanoparticles (NP+Q). After trypsinization, cells were seeded in 96-well plates at the density of 3 × 10^3^ cells/well in 100 μL of medium DMEM 10%. After 24 h incubation at 37 °C, the medium was removed and replaced with a fresh one containing increasing concentrations of NP+Q (0.6 μM, 1.2 μM, 2.5 μM, 5 μM). Cells were incubated under these conditions for a time course spanning 24 h, 48 h, and 72 h. The cell viability was assessed by MTT assay. The MTT solution (5 mg/mL in PBS) was added (10 μL/well) and the plates were incubated for 3 h at 37 °C. Then, the MTT-formazan salts were dissolved with an isopropanol/hydrochloric acid 10% solution for 20 min in constant agitation. Then, the absorbance values of the solution were measured at 570 nm using a BioRad 550 microplate reader (BioRad Laboratories, Milan, Italy). Combination index (CI) between NPs and quercetin was calculated by the dedicated software Compusyn, using dose-effect curves of the different drugs. CI values lower than 1, equal to 1, and higher than 1 indicate synergy, additivity, and antagonism, respectively. Data are as mean ± standard deviation (SD). Each value was obtained from three independent experiments.

#### 2.5.3. Statistical Analysis

All data were expressed as mean values ± SD of three independent experiments. Statistical evaluation of the data was performed by one-way ANOVA compared to the control, followed by Tukey–Kramer. Significant differences were determined at *p* value < 0.05 (*), *p* < 0.005 (**); *p* < 0.001 (***); *p* < 0.0001 (****). Graphs were obtained using GraphPad Prism Version 6 software (San Diego, CA, USA).

## 3. Results

### 3.1. Synthesis and Characterization of PCL-PEG-PCL Copolymer

**Scheme 1:**
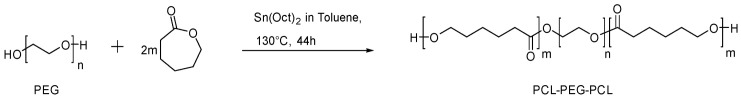
Synthesis of triblock PCL-PEG-PCL copolymer.

**Figure 1:**
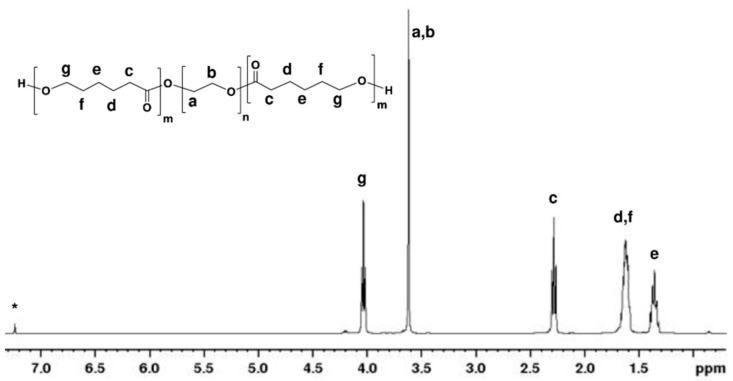
^1^H NMR spectrum (400 MHz, CDCl_3_, 298 K) of triblock PCL-PEG-PCL copolymer.

PCL and PEG are the most popular materials for the preparation of DDSs [18]. The synthesis of PCL–PEG block copolymers can be achieved by the ROP of ε-CL using a telechelic PEG diol as initiator. The amphiphilic triblock PCL-PEG-PCL copolymer was thus obtained by ROP of ε-CL with PEG diol (2000 g/mol) as initiator and Sn(Oct)_2_ as catalyst under nitrogen atmosphere (Scheme 1). The ^1^H NMR spectrum in CDCl_3_ (Figure 1) confirmed that the triblock PCL-PEG-PCL copolymer was successfully synthesized due to the presence of characteristic signals corresponding to both PCL (1.36, 1.61–1.65, 2.29, and 4.04 ppm) and PEG (3.65 ppm). In the copolymer sample, the molar ratio between the ε-CL units and the ethylene glycol units, evaluated by ^1^H NMR analysis, was 79:45. To determine the molecular weight of the triblock PCL-PEG-PCL copolymer, ^1^H NMR and GPC analysis were performed. In particular, the ^1^H NMR in CDCl_3_ showed a molecular weight of 11,020 g/mol, evaluated by integrating the -CH_2_O- peak at 4.04 ppm of PCL versus the -OCH_2_CH_2_O- peak at 3.65 of PEG. In Figure S1 the ^1^H NMR of PEG diol is reported. This value was in perfect agreement with the theoretical molecular weight (*M_n_* = 11,030 g/mol). The GPC analysis, performed using THF, indicated a molecular weight of 19,420 g/mol and a dispersity *Đ* = 1.9 (Figure S2). The GPC-chromatogram of the copolymer showed monomodal distribution of the molecular weight, thus indicating that no transesterification reaction has occurred during the ring-opening polymerization (Figure S2); moreover the molecular weight increased respect to the PEG diol (Figure S3).

In the Fourier Transform Infrared (FTIR) spectrum of PCL-PEG-PCL, shown in Figure S4, a strong C=O peak appeared at 1721 cm^−1^. In addition, the CH stretching band of caprolactone monomeric unit shifted to 2943 cm^−1^, in respect to the one of the ε-CL monomers, while the band of CH stretching vibration of PEG at 2894 cm^−1^. Furthermore, the spectrum showed a decrease in the intensity of the –OH peak (3560 cm^−1^) compared with peaks of individual PEGs and PCLs, respectively [22]. This indicated the formation of triblock PCL-PEG-PCL copolymer.

**Figure 2:**
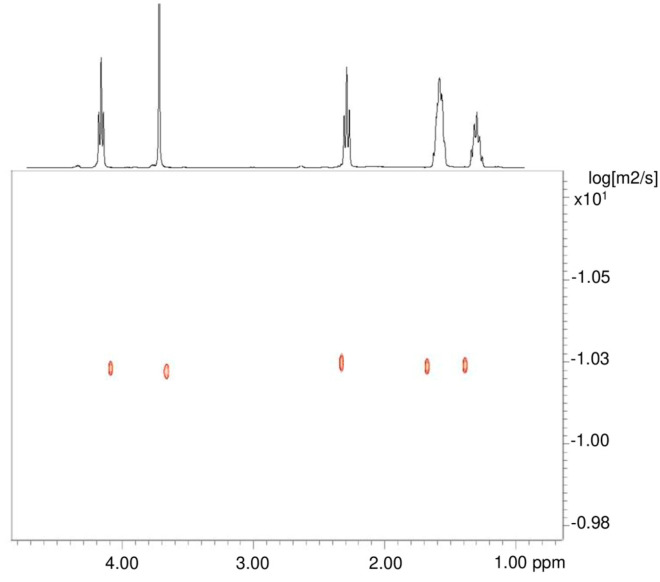
DOSY NMR spectrum (400 MHz, CDCl_3_, 298 K) of triblock PCL-PEG-PCL copolymer.

Finally, the Diffusion Ordered Spectroscopy (DOSY) NMR analysis showed that the PEG and PCL portion had the same diffusion coefficient and that they therefore belonged to the same macromolecule (Figure 2). This result clearly demonstrated that the obtained product was the desired PCL-PEG-PCL triblock copolymer and not simply the mixture of the two homo-polymers. As a reviewer suggested, for comparison purposes, the DOSY spectrum of a simple PCL sample has been registered as well (Figure S5).

### 3.2. Self-Assembly of Triblock PCL-PEG-PCL Copolymers to Form Nanoparticles

Due to its amphiphilic character, the PCL-PEG-PCL copolymers in water phase could self-assemble to form micelles, having the hydrophilic PEG as a corona and the hydrophobic PCL as a core [18]. In the hydrophobic core, a lipophilic molecule can be loaded, and the micelles may be used as carrier for, e.g., poorly water-soluble drugs. 

The micelle solutions were prepared using the dialysis method. The PCL-PEG-PCL copolymers were first dissolved in a water miscible organic solvent, such as DMF. Then the DMF copolymer solution was added to the water and spontaneous formation of micelles occurred due to reduced polymer solubility and interfacial hydrodynamic phenomena. The organic solvent was removed by dialyzing against water, and the nanoparticles (NP) were recovered by freeze-drying. 

With the same approach, nanoparticles loaded with NR (NP+NR) and quercetin (NP+Q) were also prepared. The NR is a hydrophobic, fluorescent, poorly water-soluble dye, and it was used as a model guest molecule for initial cellular study. Subsequently, quercetin was loaded into the micelles to evaluate its antioxidant and antiproliferative activity on tumor cells.

To form drug-loaded micelles, the PCL-PEG-PCL copolymer and drug were dissolved in minimum amount of DMF. This solution was added slowly into water, transferred into the dialysis bag (MWCO: 6000–8000 g/mol), and dialyzed against water for 3 days. The dialysis solution was replaced with fresh deionized water every 3 h. The so-formed micelles were dried using lyophilization. The loading of the drugs (NR or quercetin) was evaluated by ^1^H NMR analysis in DMSO (Figures S6–S8). The loading of quercetin into the nanoparticles was 1.6% *w*/*w*; the same value was obtained for the NR.

**Figure 3:**
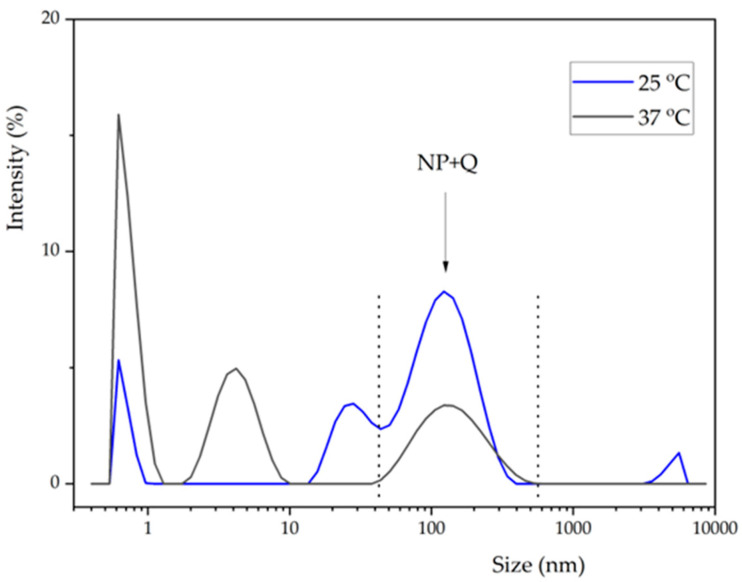
Quercetin loaded nanoparticles (NP+Q) size distribution obtained in water by DLS analysis at 25 and at 37 °C.

The quercetin-loaded nanoparticle (NP+Q) dimensions were evaluated by dynamic light scattering (DLS) at 25 °C and 37 °C (Figure 3). The data are summarized in Table 1. The average diameter of the nanoparticles was 152.5 and 128.5 nm with a PDI of 0.26 and 0.21 at 25 and 37 °C, respectively. Additional peaks (around 0.7, 4–11, and 5000 nm) are attributed to contaminants in the water and observed also in the absence of quercetin loaded nanoparticles (See Figure S9). Interestingly, at higher temperatures, narrower size distributions were observed, and the average values determined for the three measurements were much closer to each other, though within the experimental error.

**Figure 4:**
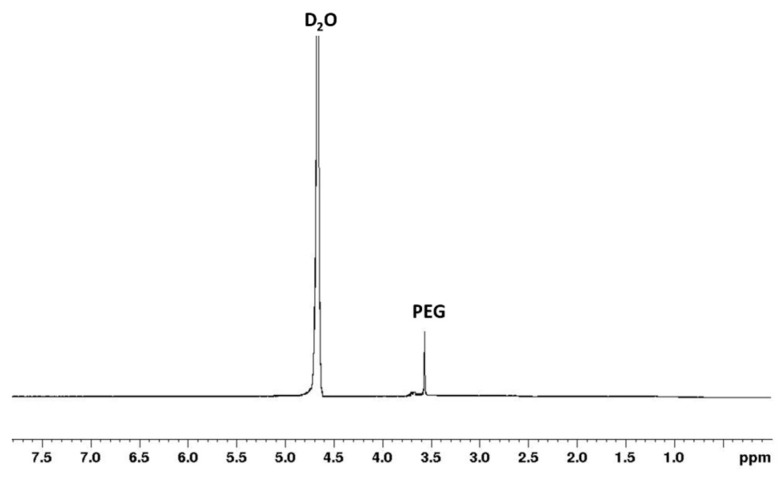
^1^H NMR spectrum (400 MHz, D_2_O, 298 K) of PCL-PEG-PCL micelles loaded with quercetin.

The ^1^H NMR spectrum in DMSO of the PCL-PEG-PCL nanoparticles (NP+Q) loaded with quercetin (Figures S8 and S10) showed the characteristic signals of quercetin (6.15, 6.37, 6.85, 7.51, 7.64, and 12.47 ppm), PEG (3.48 ppm), and PCL (1.28, 1.54–1.47, 2.22, and 3.95 ppm), while the NMR spectrum in D_2_O of the same micelles showed only the signal of the PEG (3.48 ppm) (Figure 4). This confirmed that, in an aqueous environment, PEG arranges itself as a “corona” around the PCL and the hydrophobic drug. The formation of flower-like core-shell structures for PCL-PEG-PCL copolymers was hypothesized. The core of the micelles accommodated the hydrophobic drugs via hydrophobic interactions, while the “shell” was a hydrophilic corona, made of PEG, which allowed the micelles to be dispersible in water.

### 3.3. Cellular Studies

#### 3.3.1. Nanoparticle Uptake in HCT 116 and LoVo Cells

**Figure 5:**
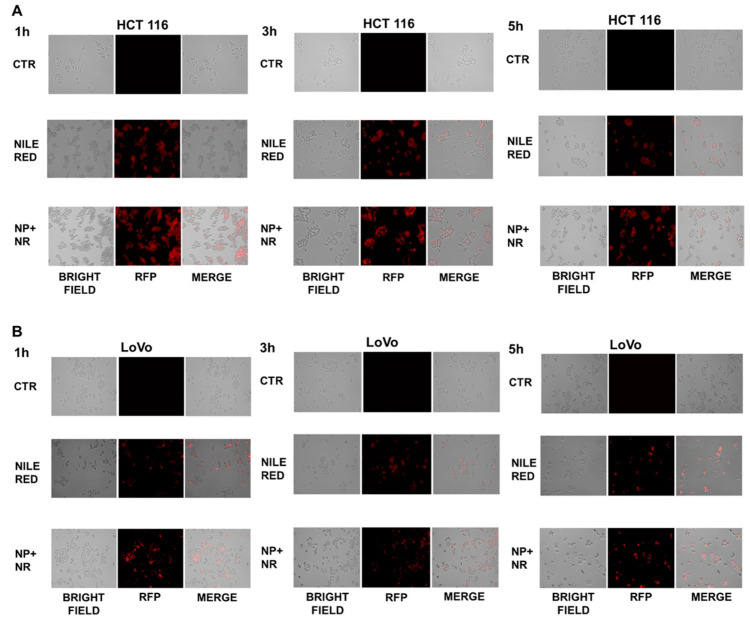
Fluorescence microscopy images of HCT 116 and LoVo, reported in panel (**A**,**B**) respectively, after 1, 3 and 5 h of incubation at 37 °C with NR and NP+NR. For each cell line, the bright field image, the red fluorescence image (RFP), and the overlap of the two images (MERGE) are reported. The cells were visualized under a fluorescence microscopy at 20× magnification.

To evaluate the cellular uptake of the nanoparticles and their intracellular distribution, fluorescence microscopy and FACS analysis were performed on HCT 116 and LoVo cells treated with Nile Red-loaded nanoparticles (NP+NR). NR is a lipophilic red dye that has been widely used to visualize and localize drug vectors [36]. The intracellular distribution of NP+NR after 1 h, 3 h, and 5 h of incubation was studied using fluorescence microscopy in both cell lines and compared to free (not loaded) NR. However, HCT 116 treated with NP+NR showed more diffuse and intense red fluorescence intensity after as early as 1 h of incubation when compared to the resulting cells incubated with free NR, and red fluorescence remained almost constant even after 3 h and 5 h of incubation (Figure 5A).

**Figure 6:**
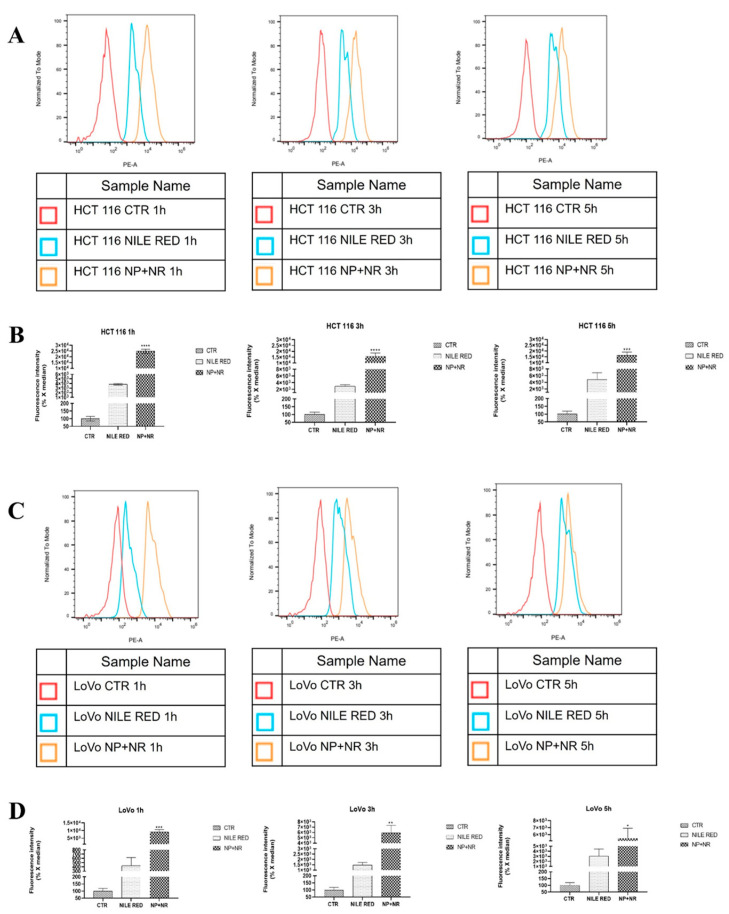
FACS analysis of nanoparticle uptake in colorectal cancer cell lines. Graphical representation of cellular NR uptake evaluated by flow cytometry in HCT 116 (**A**) and LoVo (**C**) cells after 1 h, 3 h, and 5 h of incubation, at the absorption peak at 590 nm wavelength (PE-A). Bar graphs reported the median X% of NR fluorescence intensitv in HCT 116 (**B**) and LoVo (**D**) cell lines. The experiments were performed at least three times and the results were always similar. Bars, SD. (* *p* < 0.05; ** *p* < 0.005; *** *p* < 0.001; **** *p* < 0.0001 vs. negative control).

In addition, LoVo showed a more widespread and stronger red fluorescence intensity after 1 h of incubation than cells incubated with free NR, but this slightly decreased after 3 and 5 h of incubation (Figure 5B). Furthermore, the cellular uptake efficiency of HCT 116 and LoVo cells was measured by flow cytometry. After 1 h, 3 h, and 5 h of cell incubation with NR and NP+NR, the median X of the fluorescence intensity compared to the negative control (CTR) was detected by FACS and assumed to be 100%. In detail, as shown in Figure 6A, in HCT 116 cells, the nanoparticle uptake reached a maximum peak after 1 h and it kept constant after 3 and 5 h; moreover, a 6-fold increase in fluorescence intensity was observed when cells were treated with NP+NR compared to cells treated with free NR (NP+NR 24,814.6% vs. NR 3831.9%). Similarly, as shown in Figure 6B, the highest cell uptake efficiency was also observed after 1 h for LoVo, but it slightly decreased after 3 h and 5 h; however, a fluorescence intensity rate of 9055.8% was observed for NP+NR treated cells compared to a rate of 426.9% for cells treated with free NR. These findings confirmed that PEG-PCL-PEG-based nanoparticles were able to increase and speed up NR uptake inside the cells by improving drug delivery and stability.

#### 3.3.2. Effects of Quercetin-Loaded Nanoparticles (NP+Q) on Colon Cancer Cell Line HCT 116

**Figure 7:**
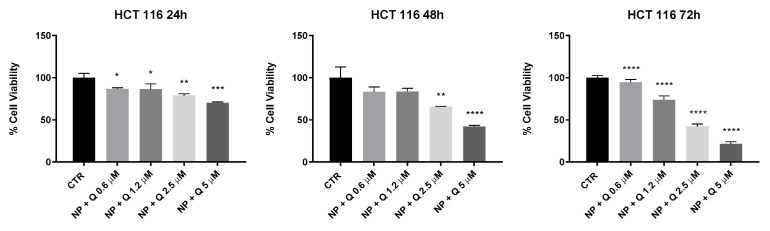
Effect of NP+Q on HCT 116 (colon cancer cell line) after 24 h, 48 h and 72 h, evaluated by MTT assay and expressed as percentage of cell viability (compared to untreated cells). Data are reported as mean of three independent experiments ± SD. (* *p* < 0.05; ** *p* < 0.005; *** *p* < 0.001; **** *p* < 0.0001 vs. negative control).

The cytotoxic effect of quercetin-loaded nanoparticles (NP+Q) was evaluated on HCT 116 cells; these cells showed a higher cellular uptake efficiency compared to LoVo by MTT assay as described in “Experimental Section”. Many studies have described the strict correlation between the dietary flavonoid intake and cancer risk; in particular, they showed that high flavonoid intake, including quercetin, could reduce the risk of colon cancer. In our colon cell model, NP+Q induced 50% growth inhibition at a concentration of 3.7 μM and 2.1 μM (IC50 values) after 48 h and 72 h, respectively (see Table 2). Interestingly, NP+Q induced a dose-dependent growth inhibition in this cell line more evident after 72 h (Figure 7). Quercetin has low stability and easily undergoes degradation; for these reasons, its antiproliferative effects are limited, with IC50 values ranging from 100 to 150 μM, as reported in previous works [37]. Our results showed that quercetin loading in PCL-PEG-PCL nanoparticles improved drug stability and efficiency; in fact, IC50 values of NP+Q indicated that the drug is already effective at low concentrations. To additionally confirm this, we compared the antiproliferative effects of NP+Q to those induced by empty NPs and free quercetin and calculated the combination index (CI) using the dedicated software Compusyn. CI values lower than 1, equal to 1, and higher than 1 indicate synergy, additivity, and antagonism, respectively. We found that CI was much lower than 1 for each tested concentration, suggesting a strong synergism between NPs and quercetin (see Figure S11).

Moreover, NP+Q were able to induce a dose dependent growth inhibition by maintaining more stable the levels of quercetin inside the nanoparticles. Quercetin is easily degraded in cell medium, as recently reported [38], and its loading in PCL-PEG-PCL nanoparticles probably enhance its antiproliferative effects by improving its stability.

## 4. Conclusions

Quercetin is a hydrophobic molecule with very poor water solubility and short blood circulation times. A critical aspect for the successful application of quercetin in cancer treatment is its encapsulation in proper delivery systems. In the realm of nanoparticles for drug delivery systems, PEG-PCL based amphiphilic copolymers are the most used and applied for in vitro cancer cells studies and for the delivery of hydrophobic drug molecules [20,21,22,23,24,25,26,27]. To the best of our knowledge, they have not been used for the delivery of quercetin into human colorectal carcinoma cells.

We have synthetized and characterized triblock ABA type PCL-PEG-PCL copolymers and used them for the preparation of nanoparticles. The triblock copolymers self-assembled in water forming micelles consisting of a core of biodegradable PCL and a corona of PEG. The copolymers formed core-shell nanoparticles and were able to incorporate quercetin into the core.

The cellular uptake efficiency of HCT 116 and LoVo cells was quantitatively determined by flow cytometry using nanoparticles loaded with NR as a model of a hydrophobic drug. The cytotoxic effect of quercetin-loaded nanoparticles (NP+Q) was evaluated on HCT 116 cells and showed promising results. Indeed, by loading the quercetin in PCL-PEG-PCL nanoparticles, its antiproliferative effects were likely enhanced by improving its stability.

Our results indicated that the prepared PCL-PEG-PCL nanoparticles are a promising DDS for the delivery of quercetin into human colorectal carcinoma cells. Further studies could be addressed to prepare different PEG/PCL nanoparticles with diverse composition [14,16,17,18,19], to evaluate the difference in nanoparticle size, loading efficiency, nanoparticle uptake, and to understand the biochemical mechanisms of quercetin uptake in carcinoma cells. Our results, moreover, encourage research in the use of PEG-PCL as a DDS not only for quercetin but also for other flavonoids, for the possible improvement of therapeutic efficacy and pharmacokinetic profiles.

## Figures and Tables

**Table 1:** DLS Data of the quercetin loaded nanoparticles (NP+Q).

Sample	Average Diameter (nm) ^1^	
NP+Q	25 °C	37 °C
	152.5 ± 77.3	128.5 ± 59.0

^1^ Determined by DLS in water.

**Table 2:** IC50 (µM) of quercetin-loaded nanoparticles on HCT 116, after 24 h, 48 h, and 72 h of treatment.

HCT 116	IC_50_
24 h	>5 µM (low toxicity)
48 h	3.7 µM
72 h	2.1 µM

## Data Availability

The data presented in this study are available on request from the authors.

## Supplementary Materials

The following supporting information can be downloaded at: https://www.mdpi.com/article/10.3390/polym15051179/s1, Figure S1: ^1^H NMR spectrum (300 MHz, CDCl_3_, 298 K) of PEG diol; Figure S2: GPC curve of triblock PCL-PEG-PCL copolymer; Figure S3: GPC curve of PEG diol; Figure S4: FT-IR spectrum of triblock PCL–PEG–PCL copolymer; Figure S5: DOSY NMR spectrum (400 MHz, CDCl_3_, 298 K) of PCL; Figure S6: ^1^H NMR spectrum (400 MHz, DMSO, 298 K) of triblock PCL-PEG-PCL copolymer; Figure S7: ^1^H NMR spectrum (300 MHz, DMSO, 298 K) of PCL-PEG-PCL micelles loaded with Nile Red; Figure S8: ^1^H NMR spectrum (400 MHz, DMSO, 298 K) of PCL-PEG-PCL micelles loaded with quercetin; Figure S9: DLS measurement of aqueous solution in the absence of quercetin loaded nanoparticles; Figure S10: ^1^H NMR spectrum (400 MHz, DMSO, 298 K) of (a) unloaded polymer, (b) quercetin and (c) polymer loaded with quercetin; Figure S11: Isobologram analysis of the effects of NP+Q combination at different concentrations.
