# Supplementary material for: Poly(ε-caprolactone)-poly(ethylene glycol) Tri-Block Copolymer as Quercetin Delivery System for Human Colorectal Carcinoma Cells: Synthesis, Characterization and In Vitro Study
Source: Polymers (Basel). 2023 Feb 26;15(5):1179. doi: 10.3390/polym15051179 (PMC10007335; doi:10.3390/polym15051179)
Supplement: Supplementary file 1 [file polymers-15-01179-s001.zip › polymers-2215332-supplementary.pdf]

SUPPORTING INFORMATION

Table of contents

Figure S1. ^1H NMR spectrum (300 MHz, $CDCl_3$, 298 K) of PEG diol…………………………………**2**

Figure S2. GPC curve of triblock PCL-PEG-PCL copolymer……………………..……………………..**2**

Figure S3. GPC curve of PEG diol…………………………………………………………………................**3**

Figure S4. FT-IR spectrum of triblock PCL–PEG–PCL copolymer……………………………..…............**3**

Figure S5. DOSY NMR spectrum (400 MHz, $CDCl_3$, 298 K) of PCL…………………………..…………...**4**

Figure S6. ^1H NMR spectrum (400 MHz, DMSO, 298 K) of triblock PCL-PEG-PCL copolymer……………..**4**

Figure S7. ^1H NMR spectrum (300 MHz, DMSO, 298 K) of PCL-PEG-PCL micelles loaded with Nile Red…………………………………………………………………………………………...……....**5**

Figure S8. ^1H NMR spectrum (400 MHz, DMSO, 298 K) of PCL-PEG-PCL micelles loaded with quercetin…………..……………......................…......................…...**6**

Figure S9. DLS measurement of aqueous solution in the absence of quercetin loaded nanoparticles………………………................…......................…......................………………………...**6**

Figure S10. ^1H NMR spectrum (400 MHz, DMSO, 298 K) of (a) unloaded polymer, (b) quercetin and (c) polymer loaded with quercetin……......................…......................……................…........................……**7**

Figure S11. Isobologram analysis of the effects of NP+Q combination at different concentrations………….**7**

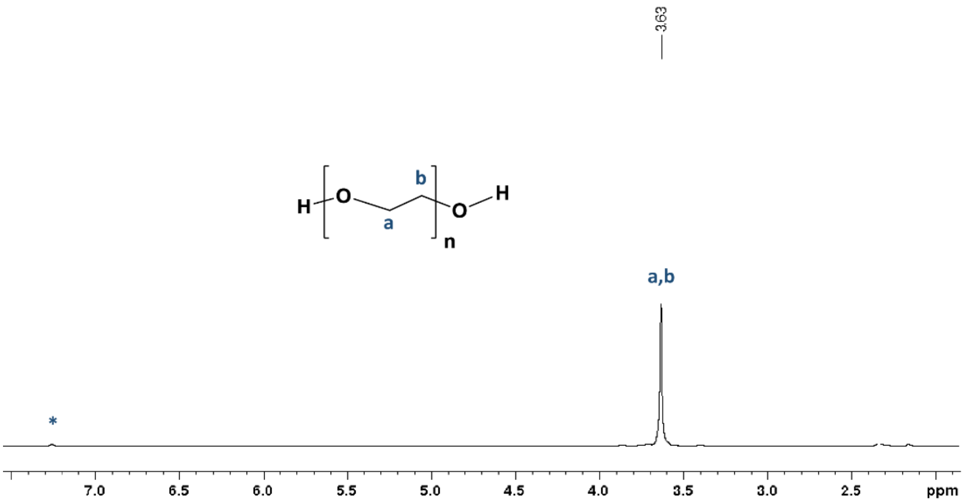

Figure S1. *^1H NMR spectrum (300 MHz, $CDCl_3$, 298 K) of PEG diol.*

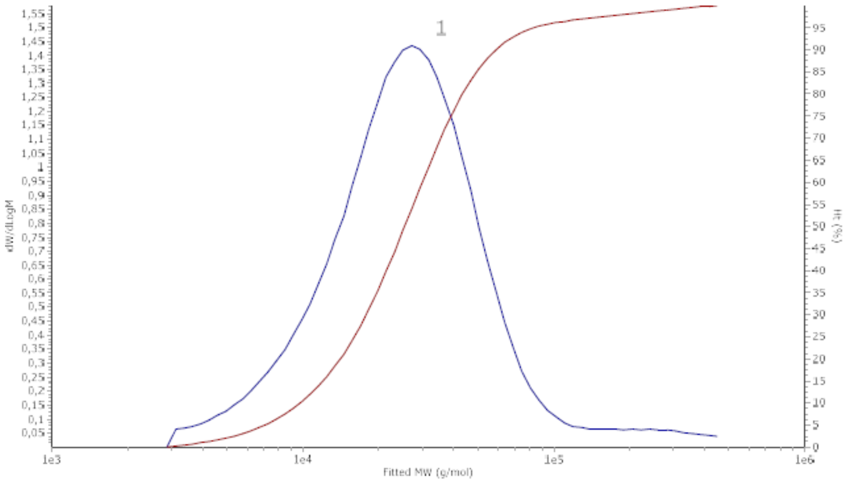

Figure S2. *GPC curve of triblock PCL-PEG-PCL copolymer.*

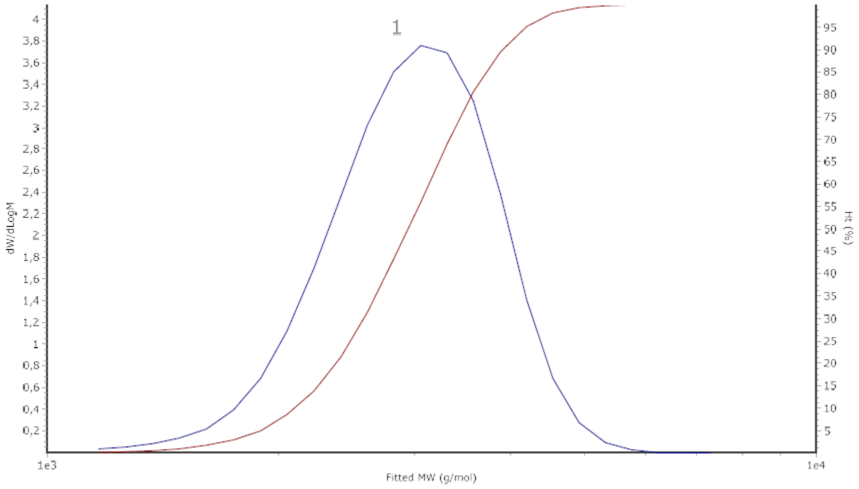

Figure S3. *GPC curve of PEG diol. GPC analysis (THF): Mn: 2870 g/mol; Đ = 1.1.*

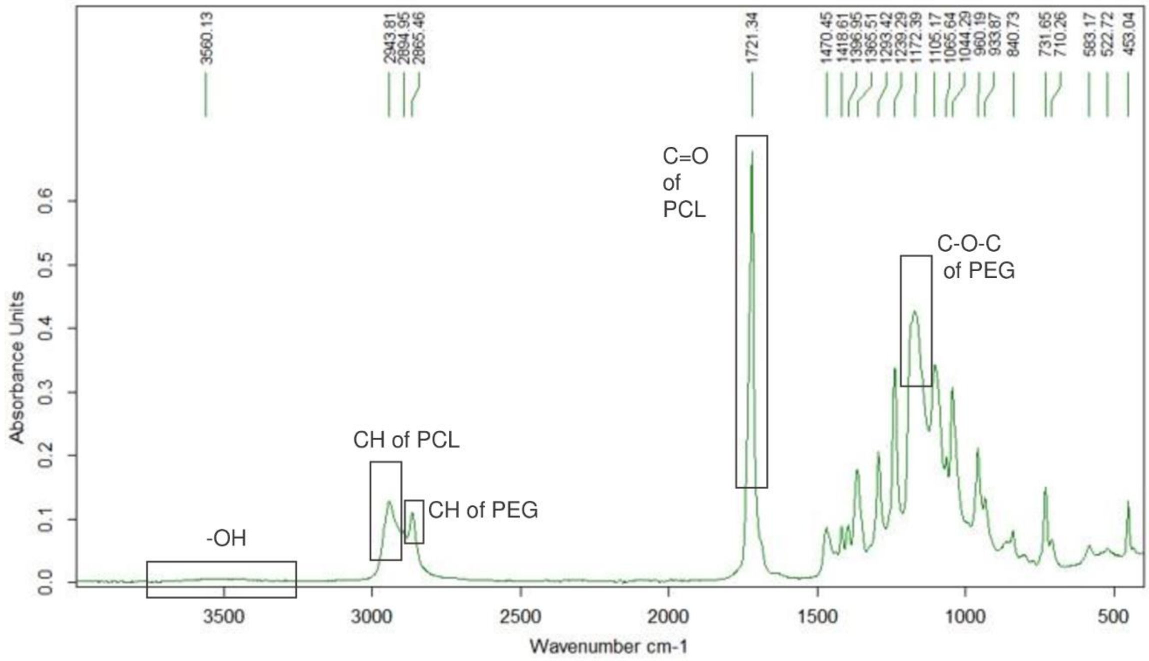

Figure S4. *FT-IR spectrum of triblock PCL–PEG–PCL copolymer.*

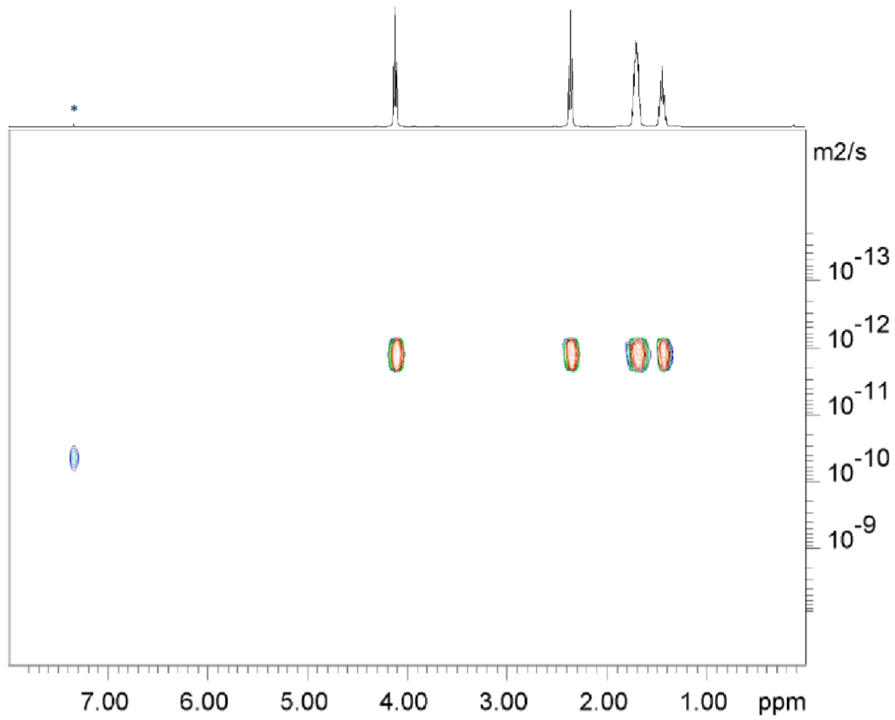

Figure S5. *DOSY NMR spectrum (400 MHz, $CDCl_3$, 298 K) of PCL.*

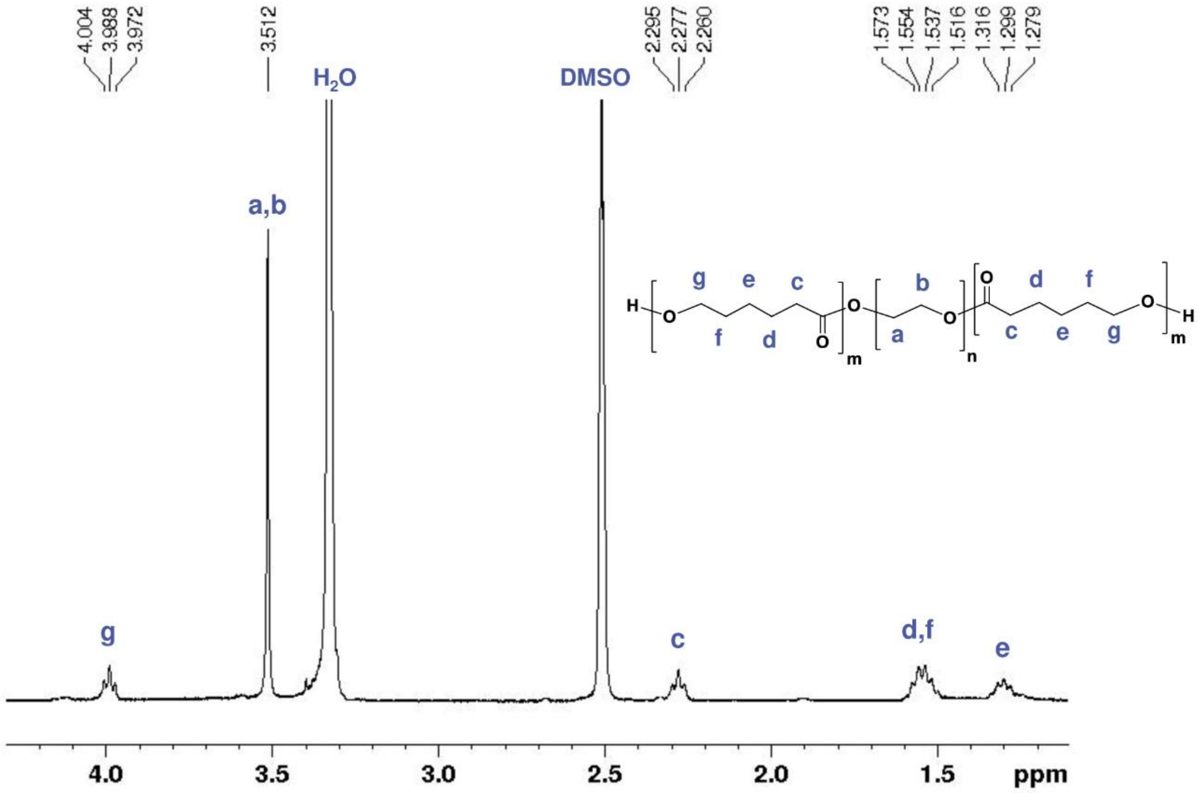

Figure S6. *^{1}H NMR spectrum (400 MHz, DMSO, 298 K) of triblock PCL-PEG-PCL copolymer.*

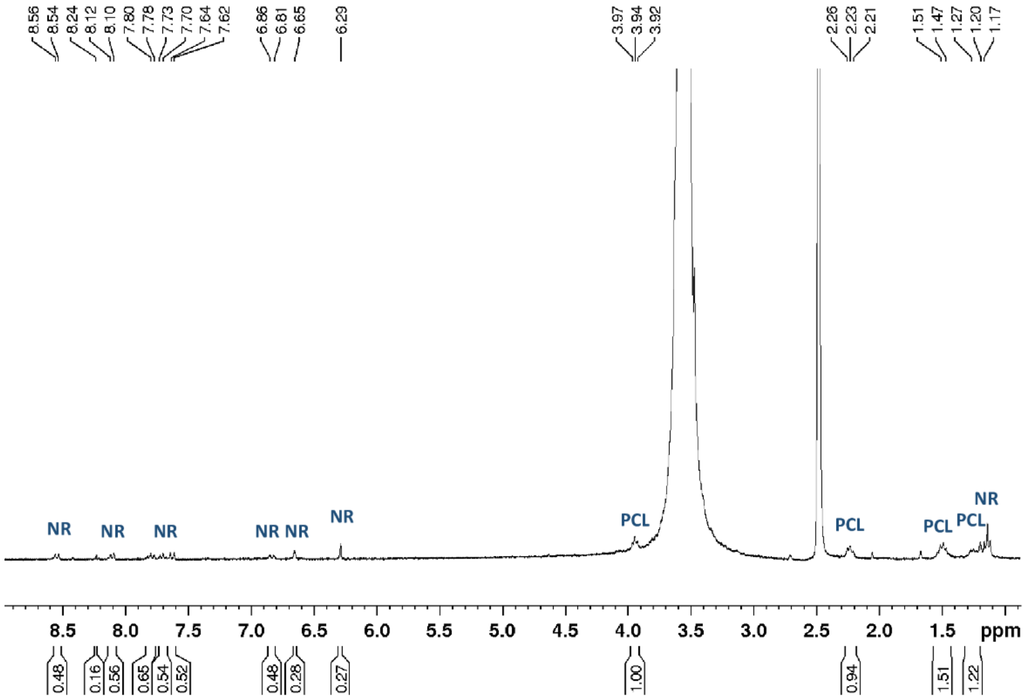

Figure S7. *^{1}H NMR spectrum (300 MHz, DMSO, 298 K) of PCL-PEG-PCL micelles loaded with Nile Red.*

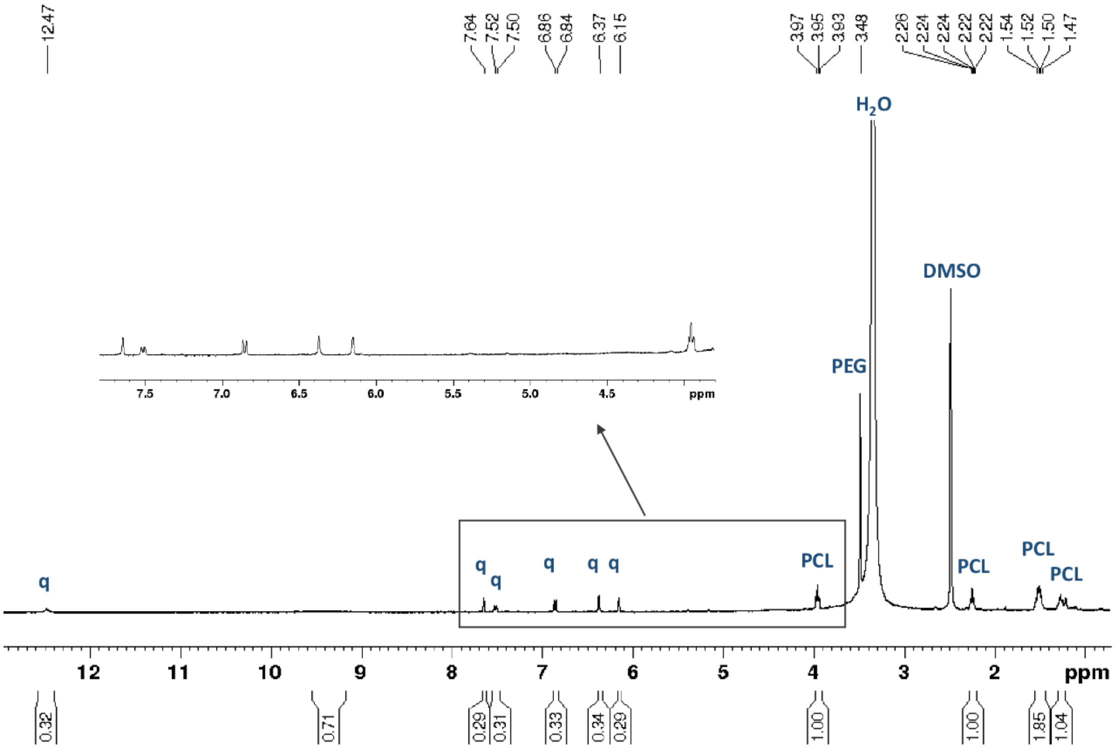

Figure S8. *^{1}H NMR spectrum (400 MHz, DMSO, 298 K) of PCL-PEG-PCL micelles loaded with quercetin.*

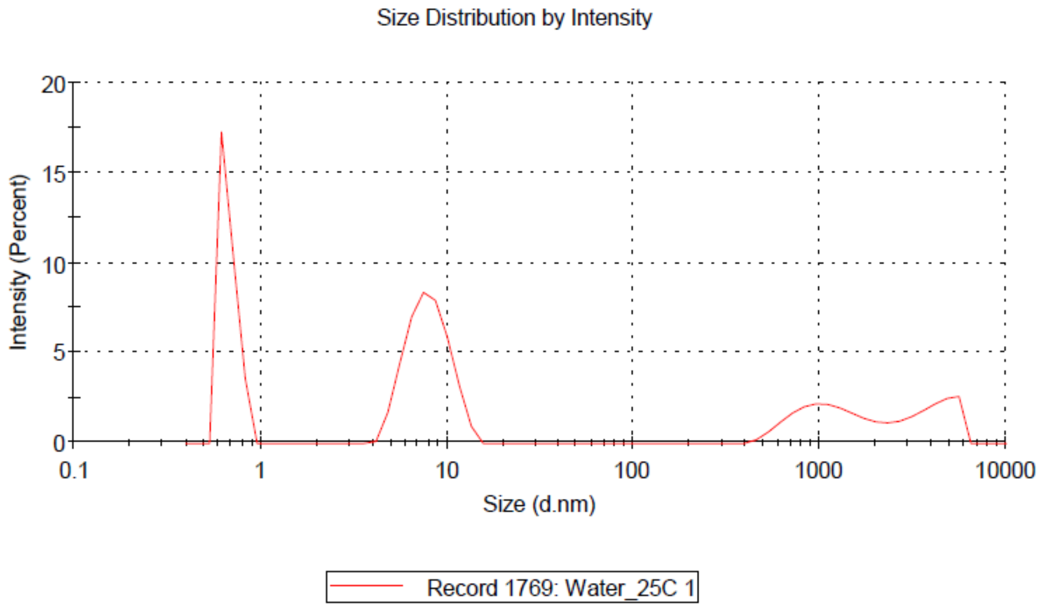

Figure S9. *DLS measurement of aqueous solution in the absence of quercetin loaded nanoparticles.*

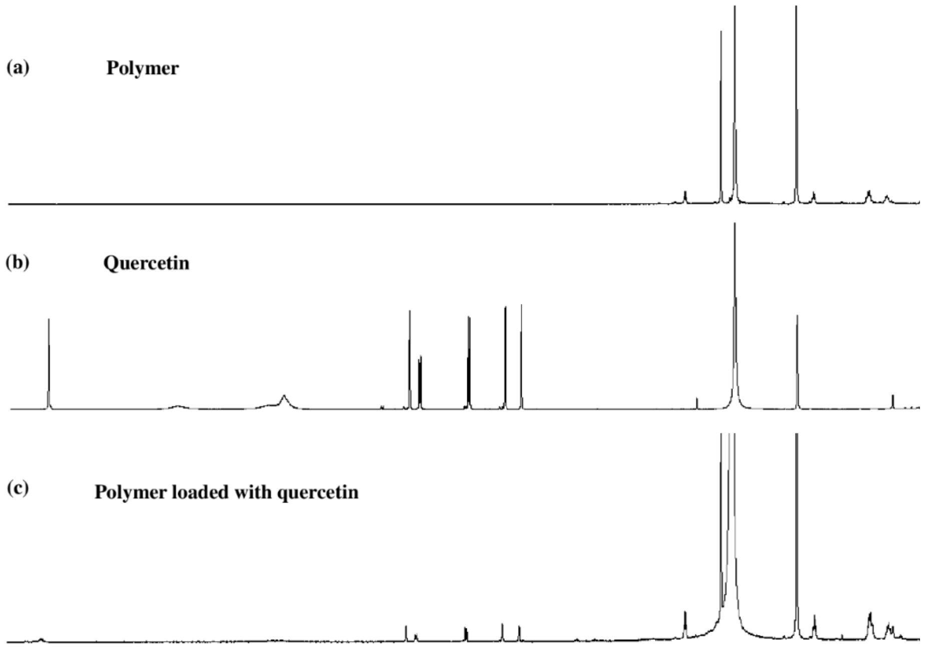

Figure S10. *^{1}H NMR spectrum (400 MHz, DMSO, 298 K) of **(a)** unloaded polymer, **(b)** quercetin and **(c)** polymer loaded with quercetin.*

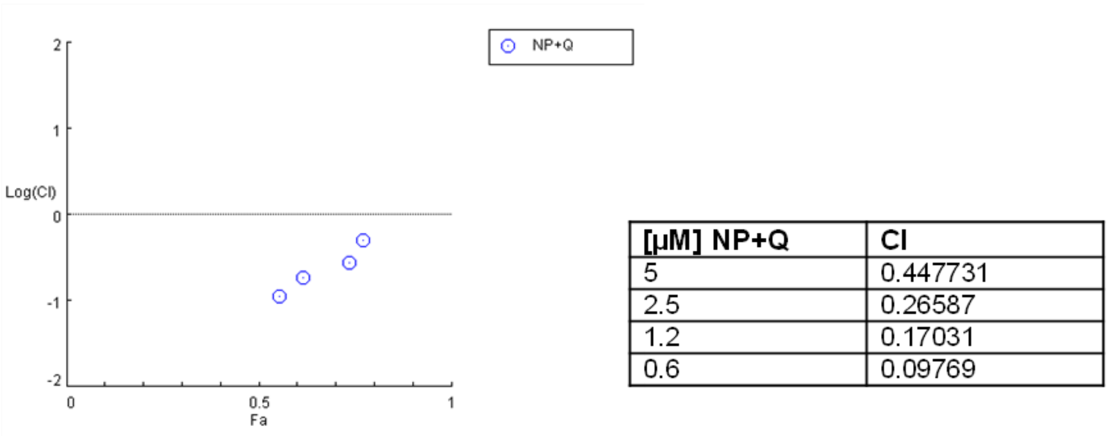

[µM] NP+Q	CI
5	0.447731
2.5	0.26587
1.2	0.17031
0.6	0.09769

Figure S11. *Isobologram analysis of the effects of NP+Q combination at different concentrations.*

Combination index between NPs and quercetin by the dedicated software Compusyn as described in Material and methods.
